# The Performance of Flash Replenishment Contrast-Enhanced Ultrasound for the Qualitative Assessment of Kidney Lesions in Patients with Chronic Kidney Disease

**DOI:** 10.3390/jcm12206494

**Published:** 2023-10-12

**Authors:** Rachel W. Walmer, Victor S. Ritter, Anush Sridharan, Sandeep K. Kasoji, Ersan Altun, Ellie Lee, Kristen Olinger, Sean Wagner, Roshni Radhakrishna, Kennita A. Johnson, W. Kimryn Rathmell, Bahjat Qaqish, Paul A. Dayton, Emily H. Chang

**Affiliations:** 1 Joint Department of Biomedical Engineering, University of North Carolina at Chapel Hill and North Carolina State University, Chapel Hill, NC 27599, USA; anush.narasimhansridharan@fccc.edu (A.S.);; 2 Department of Biostatistics, University of North Carolina at Chapel Hill, Chapel Hill, NC 27599, USA; 3 Fox Chase Cancer Center, Philadelphia, PA 19111, USA; 4 Triangle Biotechnology, Durham, NC 27709, USA; 5 Department of Radiology, University of North Carolina at Chapel Hill, Chapel Hill, NC 27599, USA; ersan_altun@med.unc.edu (E.A.); kristen_olinger@med.unc.edu (K.O.);; 6 Department of Medicine, University of North Carolina at Chapel Hill, Chapel Hill, NC 27599, USAemily_chang@med.unc.edu (E.H.C.); 7 Department of Medicine, Vanderbilt University, Nashville, TN 37232, USA

**Keywords:** chronic kidney disease, contrast-enhanced ultrasound, kidney lesions, performance metrics, screening tool

## Abstract

We investigated the accuracy of CEUS for characterizing cystic and solid kidney lesions in patients with chronic kidney disease (CKD). Cystic lesions are assessed using Bosniak criteria for computed tomography (CT) and magnetic resonance imaging (MRI); however, in patients with moderate to severe kidney disease, CT and MRI contrast agents may be contraindicated. Contrast-enhanced ultrasound (CEUS) is a safe alternative for characterizing these lesions, but data on its performance among CKD patients are limited. We performed flash replenishment CEUS in 60 CKD patients (73 lesions). Final analysis included 53 patients (63 lesions). Four readers, blinded to true diagnosis, interpreted each lesion. Reader evaluations were compared to true lesion classifications. Performance metrics were calculated to assess malignant and benign diagnoses. Reader agreement was evaluated using Bowker’s symmetry test. Combined reader sensitivity, specificity, positive predictive value (PPV), and negative predictive value (NPV) for diagnosing malignant lesions were 71%, 75%, 45%, and 90%, respectively. Sensitivity (81%) and specificity (83%) were highest in CKD IV/V patients when grouped by CKD stage. Combined reader sensitivity, specificity, PPV, and NPV for diagnosing benign lesions were 70%, 86%, 91%, and 61%, respectively. Again, in CKD IV/V patients, sensitivity (81%), specificity (95%), and PPV (98%) were highest. Inter-reader diagnostic agreement varied from 72% to 90%. In CKD patients, CEUS is a potential low-risk option for screening kidney lesions. CEUS may be particularly beneficial for CKD IV/V patients, where kidney preservation techniques are highly relevant.

## 1. Introduction

Patients with chronic kidney disease (CKD) are subject to routine kidney imaging and are especially susceptible to incidental findings. Patient-dependent management strategies include no intervention, active surveillance, ablative therapy, or surgery [1,2,3]. Incompletely characterized imaging findings often undergo further evaluation with multiphase contrast-enhanced computed tomography (CECT) or magnetic resonance imaging (CEMRI) [2,3]. Atypical lesions can be difficult to diagnose and are typically assessed by CECT or CEMRI using Bosniak criteria [3,4]. CECT and CEMRI possess high sensitivity and specificity [5,6,7,8]; however, in patients with advanced kidney dysfunction, one or both contrast agents may be contraindicated [9]. Non-contrast CT provides little information on these lesions and potential MRI techniques are limited by higher costs and lack of wide-spread accessibility, which create barriers for long-term kidney lesion management using MRI [10,11,12].

Contrast-enhanced ultrasound (CEUS) has emerged as an alternative to CECT and CEMRI. It has many attractive attributes including low cost, lack of ionizing radiation, real-time imaging capabilities, portability, accessibility, and patient tolerability, including in those with kidney dysfunction [10,12,13,14]. CEUS has the ability to characterize kidney lesions in strong agreement with CECT, the current imaging standard [13,15,16]. Reported sensitivities for CEUS are comparable to those of CECT and CEMRI, with specificity only slightly lower [6,17,18,19]. Many studies have reported high sensitivity (86–100%) and moderate to high specificity (63–86%) when using qualitative CEUS features to diagnose kidney lesions [6,17,18,19,20], and a particularly notable study by Barr et al., 2014 achieved remarkable sensitivity (100%) and specificity (96%) [21]. However, few studies have explored CEUS in patients with impaired kidney function [19,22,23].

Clinically, CEUS is used to diagnose lesions by qualitatively assessing features concerning for malignancy [6,15]. In this study, we investigated CEUS as a method to characterize solid and cystic kidney lesions in CKD patients.

## 2. Materials and Methods

### 2.1. Study Design and Participants

We conducted a cross-sectional observational study to examine CEUS diagnostic capacity for solid and cystic kidney lesions in CKD patients. This study was performed with Institutional Review Board approval (#15-1866), in accordance with ethical standards outlined in the Helsinki declaration. Written informed consent was obtained from all participants. Patients were recruited from Nephrology clinics at University of North Carolina Hospital. Patients that met study criteria were offered participation. Inclusion criteria were the following: (1) presence of kidney disease (estimated glomerular filtration rate < 90 mL/min/1.73 m^2^), presence of albuminuria or proteinuria > 30 mg/g, dialysis, an active kidney transplant, or a biopsy-proven disease; (2) one kidney lesion based on imaging in the past 6 months suspected for malignancy or warranting follow-up imaging, including Bosniak IIF cystic lesions and greater; and (3) ability to provide consent and comply with study protocol. Exclusion criteria were the following: (1) any contraindications to the contrast agent, including hypersensitivity; (2) severe pulmonary hypertension or adult respiratory distress syndrome; (3) critical illness or intensive care unit status; (4) right-to-left cardiac shunt (at the time the study was conducted, cardiac shunt was listed as a contraindication to DEFINITY^®^ Perflutren Lipid Microspheres, but this contraindication has since been removed); (5) active cardiac disease defined as severe congestive heart failure class IV NYHA, unstable angina, severe arrhythmia, myocardial infarction within 14 days, or severe hypertension (systolic > 180 mmHg or diastolic > 100 mmHg), (6) unstable neurologic disease (i.e., stroke, seizure) within 3 months; (7) invasive kidney procedure (i.e., kidney biopsy, non-surgical cytoreductive procedure) between lesion identification and CEUS; (8) medical condition that would decrease data reliability (i.e., mental illness, drug abuse); (9) pregnancy or lactation; and (10) obesity limiting acquisition of quality images. After obtaining informed consent, patients underwent CEUS per a standardized study protocol using flash replenishment imaging (Supplemental Figure S1). For patients with multiple lesions, the most concerning lesion by prior imaging was evaluated with CEUS. If multiple lesions were present, met criteria, and consent was given, then multiple lesions were imaged as separate cases.

### 2.2. Imaging Protocol

**Figure 1:**
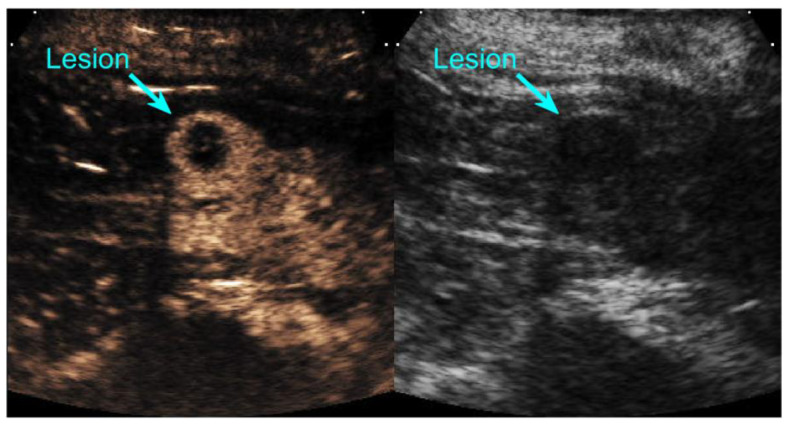
Dual split-screen ultrasound image of a kidney lesion in contrast mode (**left side**) and B-mode (**right side**). The lesion is labeled with a cyan blue arrow.

Ultrasound (US) images were acquired by registered sonographers using a Siemens Acuson Sequoia 512 (Siemens, Mountain View, CA, USA) and a curvilinear abdominal transducer (1–4 MHz) oriented over the lesion and part of the kidney parenchyma. Lesions were located using B-mode US at a standard mechanical index (MI = 1.9). CEUS imaging used a low MI Cadence Pulse Sequencing (CPS) mode to limit contrast disruption during acquisition. CPS MI (0.18), dynamic range (80 dB), capture rate (10 Hz), flash duration (<1 s) and frequency (1.5 MHz) were kept constant across patients. Gain, depth, and infusion rate were optimized per patient. Flash sequences used acceptable MI ranges (0.7–0.8 depending on depth) for Perflutren Lipid Microspheres (DEFINITY^®^, Lantheus Medical Imaging, North Billerica, MA, USA). A 50 mL saline solution mixed with 1.3 mL of Perflutren Lipid Microspheres was infused at 4, 6, or 8 mL/min based on patient body mass indices <21, 21–30, or >30 kg/m^2^, respectively, using a Medfusion^®^ 4000 syringe pump (Smiths Medical, Minneapolis, MN, USA). Two flash replenishment clips and a sagittal kidney sweep were collected in either CPS only or split-screen CPS and B-mode imaging (Figure 1, Supplemental Video S1).

### 2.3. Image Preparation and Reader Interpretation

Cases were de-identified and stripped of clinical information before being interpreted by four readers using a custom user interface (UI) (Supplemental Figure S2) developed in MATLAB^®^ (Mathworks, Natick, MA, USA). Readers were radiologists with 0.25–3 years of experience interpreting CEUS images. Prior to interpretation, readers reviewed UI operating instructions and viewed a lecture on kidney CEUS imaging [24]. Bosniak criteria for cystic lesions were modified for CEUS (Table 1) and used by readers to classify lesions as benign, malignant, or indeterminate. One flash replenishment clip and sweep per case were provided to evaluate lesions.

### 2.4. Reference Standards for Lesion Diagnostics

True lesion diagnosis was confirmed by tissue pathology or follow-up imaging when pathology was unavailable. The follow-up interval (≥12 months) and modality (US, CT, MRI) were determined by the patient’s clinician. The most recent exam was used when patients had multiple follow-up examinations. Pathology samples were either malignant or benign for disease. Follow-up imaging diagnoses were (1) stable for lesions with no change, regression, or no concerning features, (2) suspicious for lesions with stable, but persistent concerning features, or (3) progressed for lesions with progression of concerning features. Suspicious lesions were likely malignant, but the patient and treatment team opted for active surveillance. Concerning characteristics included enhancing or thickened septations, calcifications, mural thickness, irregularity, nodules, or solid enhancing masses.

### 2.5. Performance Measures and Statistical Analysis

CEUS diagnostic accuracy for characterizing malignant and benign lesions was evaluated by comparing reader interpretations to true lesion diagnoses using the full lesion dataset, only cystic lesions, and by grouping lesions according to CKD stage. Groups were CKD II/III (*n* = 31; II: 10, III: 21), CKD IV/V (*n* = 17; IV: 10, V: 7), and end-stage kidney disease (ESKD, *n* = 15). Diagnostic performance was assessed through sensitivity, specificity, positive predictive values (PPV), and negative predictive values (NPV) for individual and combined reader interpretations. Combined reader measures were estimated using functions of the logistic regression parameters fitted via Generalized Estimating Equations to account for the correlation of measurements made on the same patient [25]. Reader agreement regarding malignant diagnoses was evaluated using Bowker’s symmetry test. A two-sided *p*-value of 0.05 was considered statistically significant; *p*-values closer to one indicated greater agreement between readers. Analysis was completed using the open-source R statistical software environment v4.0 and SAS v9.4 (Cary, NC, USA).

**Figure 2:**
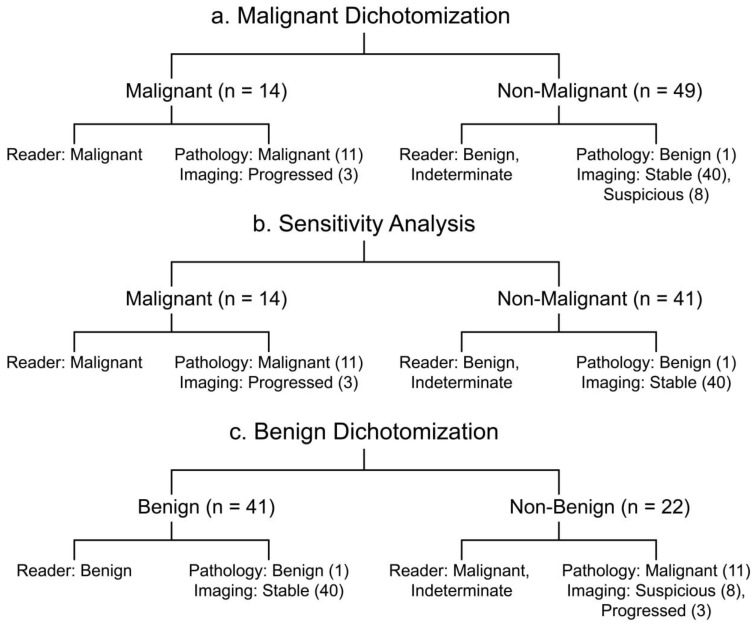
Dichotomization of reference standards and reader diagnoses by (**a**) malignancy, (**b**) sensitivity analysis, and (**c**) benignity.

Reader interpretations and true values were first dichotomized by malignancy status (Figure 2). Malignant reader ratings, malignant pathology, and progressed lesions per follow-up imaging were considered malignant. Benign or indeterminate reader ratings, benign pathology, and stable or suspicious lesions per follow-up imaging were considered non-malignant. For sensitivity analysis, we repeated this dichotomization after excluding suspicious cases (Figure 2). Results were then dichotomized based on benignity (Figure 2). Benign reader ratings, benign pathology, and stable lesions per follow-up imaging were considered benign. Malignant or indeterminate reader ratings, malignant pathology, and suspicious or progressed lesions per follow-up imaging were considered non-benign.

## 3. Results

### 3.1. Disease Severity, Lesion Diagnoses, and Reader Agreement

**Figure 3:**
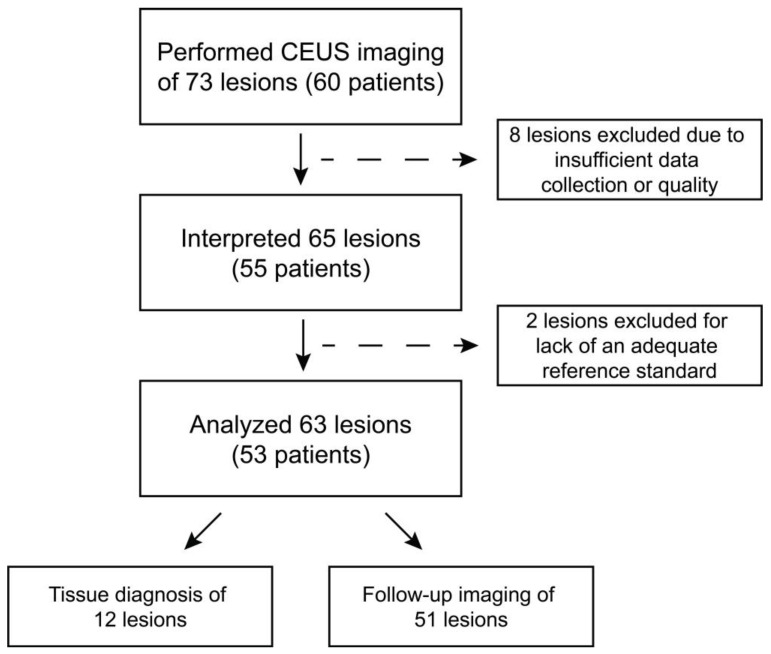
Flow diagram of data retention from imaging through final analysis.

Overall, 73 lesions (60 patients) were imaged and 63 lesions (53 patients) were included in the final analysis after excluding 10 lesions due to data inadequacies (Figure 3). Among included patients, disease severity ranged from CKD II to ESKD; CKD III (17/53, 32.1%), and ESKD (14/53, 26.4%) were most common (Table 2). Patient characteristics, CKD stage, and initial imaging study for patients included in final analyses (Table 2) and all enrolled patients (Supplemental Table S1) were reported. Tissue pathology was available for 12 lesions and follow-up imaging for 51 lesions. Information on analyzed lesions was provided in Supplemental Table S2. Inter-reader agreement regarding lesion malignancy varied from 72% to 90% (Supplemental Table S3). Reader agreement *p*-values were 0.2, 0.3, and 0.4 for Reader 1 vs. 2, 3, and 4, respectively; 0.7 and 0.8 for Reader 2 vs. 3 and 4, respectively; and 0.6 for Reader 3 vs. 4 (Supplemental Table S3).

### 3.2. Diagnostic Performance for Malignant Lesions

In total, 14 lesions were malignant and 49 were non-malignant (Figure 2). Combined reader sensitivity, specificity, PPV, and NPV for diagnosing malignant kidney lesions were 71%, 75%, 45%, and 90%, respectively. Individual readers achieved a 64–79% sensitivity, a 71–78% specificity, 42–50% PPVs, and 88–93% NPVs (Table 3). With sensitivity analysis, individual reader specificity (85–88%) and PPV (60–69%) improved (Table 3). Combined sensitivity, specificity, PPV, and NPV were 71%, 87%, 65%, and 90%, respectively. Considering only cystic lesions, combined reader sensitivity, specificity, PPV, and NPV were 60%, 87%, 52%, and 90%, respectively. Specificity and PPV increased after performing sensitivity analysis (Table 4). CEUS was sensitive to all solid malignant lesions. By grouped CKD stage, combined reader sensitivity was 71%, 81%, and 58% for CKD II/III, CKD IV/V, and ESKD data, respectively. Combined specificity was 68%, 83%, and 81% for CKD II/III, CKD IV/V, and ESKD data, respectively. Specificity and PPV increased after performing sensitivity analysis in this group (Table 4).

### 3.3. Diagnostic Performance for Benign Lesions

Overall, 41 lesions were benign and 22 were non-benign (Figure 2). For diagnosing lesion benignity, combined reader sensitivity, specificity, PPV, and NPV were 70%, 86%, 91% and 61%, respectively. Individual readers achieved a 63–80% sensitivity, a 82–91% specificity, 88–93% PPVs, and 56–70% NPVs (Table 3). For only cystic lesions, combined reader sensitivity, specificity, PPV, and NPV were 75%, 79%, 90%, and 54%, respectively (Table 4). Combined reader sensitivity by grouped disease stage was 67%, 81%, and 63% for CKD II/III, CKD IV/V, and ESKD, respectively. Combined specificity was 85%, 95%, and 80% for CKD II/III, CKD IV/V, and ESKD grouped data, respectively (Table 4). The CKD IV/V group had the highest PPV (98%) when diagnosing lesion benignity (Table 4).

## 4. Discussion

This study evaluated CEUS diagnostic accuracy for suspicious cystic and solid kidney lesions in CKD patients. We showed that flash replenishment CEUS could be used to qualitatively characterize kidney lesions with moderately high sensitivity and specificity (Table 3). CEUS performed best in CKD IV/V patients (Table 4), and our high NPV (90%) showed that CEUS can confidently identify benign lesions.

Previous studies have compared the sensitivity of CEUS for diagnosing complex cystic kidney lesions to CECT, CEMRI, and unenhanced ultrasound. Quaia et al., 2008 demonstrated that CEUS had a diagnostic sensitivity between 86% and 95% for three different readers, while unenhanced ultrasound only achieved a sensitivity between 43% and 48% for those same readers [17]. Similarly, Xue et al., 2014 found that the diagnostic concordance between CEUS and pathology versus unenhanced ultrasound and pathology were significantly different: CEUS had an 88.3% agreement with pathology while unenhanced ultrasound only had a 59.2% agreement [18]. Chen et al., 2015 determined that CEUS was more sensitive to complex lesions compared to CEMRI, albeit slightly less specific [6]. Additionally, Tufano et al., 2022 recently demonstrated that quantitative parameters such as peak intensity and area under the curve could be used to characterize renal masses with high accuracy: 93% and 95%, respectively [20]. However, the current literature reports few studies using CEUS to characterize kidney lesions in patients with kidney dysfunction. Prior studies have included a small subset of patients with kidney insufficiency [19,23], with one study comparing diagnostic performance in patients with and without CKD [22]. High accuracy (>89%) was reported for determining lesion pathology in the presence of abnormal kidney function, but differences in study design, assessment criteria, and final reported metrics make direct comparison to our outcomes challenging [19,23]. High sensitivity (86–100%) and moderately high specificity (63–86%) have been reported using CEUS [6,17,18,19,21]. In CKD patients specifically, high sensitivity (96%) and moderate specificity (50%) were reported [22]. In comparison, here, we achieved a lower combined sensitivity for identifying malignant lesions (71%) but a specificity (75%) comparable to previous values. Here, sensitivity was likely reduced due to the lower percentage of malignant lesions in this study (22.2%, 14/63) compared to prior results (52.3%, 23/44).

We performed sub-analyses to further investigate CEUS performance. Specificity for characterizing benign lesions was higher than for malignant lesions, indicating more true negative and less false positive results through benign dichotomization, i.e., only classifying as benign those without suspicious features. PPV also increased, suggesting a decrease in false positive results with benign over malignant criteria. By grouped CKD stage performance metrics were notably highest for CKD IV/V (sensitivity: 81%, specificity: 83%) when determining lesion malignancy. When characterizing benignity, CKD IV/V had a sensitivity, specificity, and PPV of 81%, 95%, and 98%, respectively. The superior performance of CEUS in CKD IV/V patients with kidney lesions compared to patients with other CKD stages suggests that CEUS could be utilized as a diagnostic tool in this patient population. This is fortuitous as kidney preservation is an important consideration in CKD IV/V patients and the use of CECT/CEMRI can sometimes be limited in patients at this stage. As such, CEUS could play a key role in safely diagnosing kidney lesions in patients with deteriorating kidney function.

Challenges with accurately characterizing kidney lesions resulted from decreased CEUS sensitivity to malignant lesions in ESKD patients [22] (Table 4), and numerous false positive results when evaluating lesion malignancy. The latter could be from readers classifying 75–100% of suspicious lesions (per follow-up imaging) as malignant, which was considered a misclassification by our malignancy dichotomization. This inflated false positive results, and removing suspicious lesions from malignancy dichotomization (sensitivity analysis) increased specificity (75% to 87%) and PPV (45% to 65%). In practice, CECT/CEMRI would label these lesions suspicious for malignancy, albeit stable over one year. This is consistent with the indolent nature of many kidney cancer subtypes, especially in CKD patients. Therefore, these cases could represent true malignancies, aligning with reader interpretations; however, this would need to be confirmed by tissue pathology. At a minimum, reader CEUS interpretations of these suspicious lesions would agree with CECT/CEMRI interpretation and should not alter clinical management.

Lack of experience reading CEUS images was a limitation in this study and may be partially responsible for decreases in performance. Reader experience interpreting CEUS images ranged from 0.25 to 3 years. This and minimal CEUS training likely disadvantaged less experienced readers, influencing their ability to accurately evaluate kidney lesions with CEUS. We would expect reader accuracy to improve with additional training and experience [17], but accuracy is also affected by the Bosniak criteria, namely difficulties differentiating Bosniak II/IIF and III lesions [4,5,7,26].

Contrast imaging modalities use CT Bosniak criteria to interpret cystic lesions [22,27]. CEUS studies characterizing kidney lesions either directly translate Bosniak criteria to CEUS features [22,27] or create parallel classification schemes based on lesion enhancement [19,21] and vascular characteristics [17,18]. Our criteria are analogous to reported CEUS criteria, incorporating septal enhancement, nodular enhancement, solid components, and thickening as important features for determining lesion malignancy (Table 1). Unlike reported methods, we implemented a binary classification scheme to compare reader designations with true values (Figure 2). This approach reduced trinary reader and surveillance imaging classifications, decreasing overall performance. Sensitivity analysis demonstrated that this was a flaw in the dichotomization approach, which could be addressed by using a different classification scheme. Lack of standardized CEUS Bosniak criteria poses a challenge with regards to selecting the optimal method to improve diagnostic power. Alone, CT Bosniak criteria insufficiently describe CEUS vascular features. This, the growing role of CEUS for diagnosing suspicious kidney lesions, and the success of MRI-specific Bosniak criteria [7,26] and CEUS-specific LI-RADS criteria for diagnosing HCC [28,29] demonstrate the value of developing standardized CEUS-specific Bosniak criteria to improve performance while minimizing the impact of other factors such as reader experience.

This study was limited by the small number of available pathology results (*n* = 12). Surveillance imaging was used when pathology was unavailable, but lesion characterization was less definitive by imaging. This study also suffered from a small number of malignant lesions (*n* = 14), affecting sensitivity and specificity [30]. Imaging technique may have presented a challenge: not all cases were acquired using dual mode imaging, causing minor misalignment between the supplied B-mode image and corresponding CEUS video data. To avoid lesion misidentification, a marker was placed on the B-mode image around the lesion of interest. In the future, always using dual mode imaging would reduce potential misalignment between images. To standardize imaging, sonographers followed a protocolized technique; however, feedback from readers (not formally analyzed) suggested that in some cases, multiple views of the lesion (typically available clinically) or an increase in contrast dose might have increased their diagnostic confidence (Supplemental Table S4). Additionally, 10 of the 73 exams could not be interpreted, two due to lack of an adequate reference standard and eight (one CKD II, two CKD III, and five ESKD) due to insufficient quality.

Lastly, conventionally, bolus imaging is repeated as necessary to observe and fully capture lesion dynamics [18,19,21]. However, we qualitatively assessed lesion malignancy using flash replenishment images, which can be acquired rapidly (10–30 s), reproducibly, and efficiently [31]. One flash replenishment infusion offers minutes of continuous imaging for repeated measures at multiple tissue locations, performing volumetric imaging, and capturing tissue heterogeneity. In contrast, one bolus dose spans multiple minutes and requires a wait time between subsequent acquisitions. Despite these advantages, flash replenishment imaging is not utilized frequently for qualitative lesion assessment. Lack of clinical familiarity with this technique may have impacted performance. To improve lesion interpretation, flash replenishment imaging could be further optimized or could be modified to capture infusion wash-in and wash-out, mimicking bolus techniques.

## 5. Conclusions

For CKD patients, CEUS is emerging as a useful tool. Developing CEUS-specific Bosniak criteria and further exploring flash replenishment imaging to characterize kidney lesions may improve diagnostic power. Our results demonstrate the potential of this modality for managing kidney lesions in patients with kidney dysfunction. Reasonable sensitivity and specificity were achieved by readers in this study, and most promising was the high NPV (90%) when assessing lesion malignancy, correspondingly high PPV (91%) for lesion benignity, and the diagnostic performance in CKD IV/V patients. To reduce surgical overtreatment [8,26,32], recent research favors conservative management of kidney lesions, even potential malignancies since some kidney cancer subtypes are slower growing and often indolent [1,26,32]. This is especially true for CKD patients, where kidney preservation is crucial. The result is a shift towards more surveillance and less intervention, particularly with smaller lesions [1,33]. Further, some kidney cysts, such as Bosniak IIF lesions, may require multiple examinations to diagnose. Reducing the cost, patient discomfort, and exposure to radiation are important considerations when repeat imaging is required [9,13]. The low cost, safety, accessibility, and portability of CEUS all favor this technology for the long-term management of kidney lesions, particularly in patients with few other imaging options.

## Figures and Tables

**Table 1:** Comparison of modified CEUS cystic mass classification with CT Bosniak criteria.

Stage	CEUS Cystic Mass Classification	Bosniak Criteria
I	Cystic mass with no enhancement	Simple cyst with hairline-thin wall No septa, calcifications, or solid components Water attenuation, no enhancement
II	Thin smooth septation or septations (less than 2 mm in thickness) with constant mild enhancement or occasional foci of enhancement	Septa: few hairline thin, but no measurable enhancement. Calcifications: fine or a short segment of slightly thickened present in wall or septa. High attenuation: uniform in lesions (<3 cm) that are sharply marginated, no enhancement.
IIF	Septation(s) between 2 and 3 mm in thickness with enhancement	Septa: multiple hairline thin, but no measurable enhancement of septum or wall. Minimal thickening of wall or septa; may contain thick and nodular calcification, but no measurable contrast enhancement. No enhancing soft tissue components. Intrarenal: totally intrarenal non-enhancing high-attenuating renal lesions, lesions are generally well marginated.
III	Cystic mass with thick (more than 3 mm in thickness) and nodular septation(s) with enhancement	Measurable enhancement: cystic mass with thickened, irregular, or smooth walls or septa; measurable enhancement present.
IV	Cystic mass with enhancing solid tissue component	Enhancing soft tissue components: clearly malignant, cystic masses that can have all criteria from Category III, but also contain distinct enhancing soft tissue components independent of wall or septa.

**Table 2:** Characteristics of patients included in the final analysis.

Patient Characteristics (*N* = 53)	*N* (%)
**Age (years)**	
Mean ± SD	60 ± 14
**Sex**	
Male	33 (62.3%)
Female	20 (37.7%)
**Race or Ethnicity**	
Black	26 (49.1%)
White	27 (50.9%)
**CKD Stage**	
CKD II	8 (15.1%)
CKD III	17 (32.1%)
CKD IV	10 (18.9%)
CKD V	4 (7.5%)
ESKD	14 (26.4%)
**Initial Imaging Study**	
Non-contrast CT	3 (5.6%)
Contrast CT	2 (3.8%)
Contrast CT with renal mass protocol	10 (18.9%)
Non-contrast MRI	3 (5.6%)
Contrast MRI	3 (5.6%)
Conventional US	32 (60.4%)

Abbreviations: CKD (chronic kidney disease), ESKD (end-stage kidney disease), CT (computed tomography), MRI (magnetic resonance imaging), US (ultrasound).

**Table 3:** Performance analysis of CEUS for diagnosing malignant kidney lesions.

Diagnostic Performance	Reader 1	Reader 2	Reader 3	Reader 4	Overall
**Diagnosing malignant lesions ^1^**					
Sensitivity (95% CI)	79% (49%, 95%)	71% (42%, 92%)	71% (42%, 92%)	64% (35%, 87%)	71% (54%, 89%)
Specificity (95% CI)	78% (63%, 88%)	76% (61%, 87%)	71% (57%, 83%)	76% (61%, 87%)	75% (64%, 86%)
PPV (95% CI)	50% (28%, 72%)	45% (24%, 68%)	42% (22%, 63%)	43% (22%, 66%)	45% (26%, 64%)
NPV (95% CI)	93% (80%, 98%)	90% (77%, 97%)	90% (76%, 97%)	88% (74%, 96%)	90% (82%, 98%)
**Sensitivity analysis ^2^**					
Sensitivity (95% CI)	79% (49%, 95%)	71% (42%, 92%)	71% (42%, 92%)	64% (35%, 87%)	71% (54%, 89%)
Specificity (95% CI)	88% (74%, 96%)	88% (74%, 96%)	85% (71%, 94%)	85% (71%, 94%)	87% (77%, 96%)
PPV (95% CI)	69% (41%, 89%)	67% (38%, 88%)	62% (35%, 85%)	60% (32%, 84%)	65% (43%, 86%)
NPV (95% CI)	92% (79%, 98%)	90% (76%, 97%)	90% (76%, 97%)	88% (73%, 96%)	90% (82%, 98%)
**Diagnosing benign lesions ^3^**					
Sensitivity (95% CI)	80% (65%, 91%)	63% (47%, 78%)	68% (52%, 82%)	68% (52%, 82%)	70% (59%, 81%)
Specificity (95% CI)	86% (65%, 97%)	86% (65%, 97%)	91% (71%, 99%)	82% (60%, 95%)	86% (76%, 97%)
PPV (95% CI)	92% (78%, 98%)	90% (73%, 98%)	93% (78%, 99%)	88% (71%, 96%)	91% (83%, 99%)
NPV (95% CI)	70% (50%, 86%)	56% (38%, 73%)	61% (42%, 77%)	58% (39%, 75%)	61% (45%, 76%)

^1^ Lesions were assessed as malignant versus non-malignant (*N* = 63). Lesions with malignant pathology (*n* = 11) or follow-up imaging labeled progressed (*n* = 3) were classified as malignant. Lesions with benign pathology (*n* = 1) or labeled stable (*n* = 40) or suspicious (*n* = 8) by follow-up imaging were classified as non-malignant. ^2^ Lesions were re-assessed as malignant versus non-malignant (*n* = 55) after removing lesions labeled suspicious by follow-up imaging (*n* = 8). ^3^ Lesions were assessed as benign versus non-benign (*N* = 63). Lesions with benign pathology (*n* = 1) or labeled stable by follow-up imaging (*n* = 40) were considered benign. Lesions with malignant pathology (*n* = 11) or labeled suspicious (*n* = 8) or progressed (*n* = 3) by follow-up imaging were classified as non-benign. Refer to method Section 2.5 *Performance Measures and Statistical Analysis* for more details on diagnostic performance analyses. Abbreviations: PPV (positive predictive value), NPV (negative predictive value), CI (confidence interval).

**Table 4:** Performance analysis of CEUS for diagnosing malignant and benign lesions by grouped disease stage and for cystic lesions.

Diagnostic Performance	CKD II/III (*n* = 31)	CKD IV/V (*n* = 17)	ESKD (*n* = 15)	Cystic (*n* = 51)
**Diagnosing malignant lesions**				
Sensitivity (95% CI)	71% (46%, 97%)	81% (49%, 113%)	58% (23%, 94%)	60% (39%, 81%)
Specificity (95% CI)	68% (50%, 85%)	83% (63%, 102%)	81% (64%, 99%)	87% (78%, 96%)
PPV (95% CI)	39% (14%, 65%)	59% (20%, 99%)	44% (2%, 85%)	52% (27%, 78%)
NPV (95% CI)	89% (77%, 101%)	93% (81%, 106%)	89% (73%, 104%)	90% (82%, 98%)
**Sensitivity analysis**				
Sensitivity (95% CI)	71% (46%, 97%)	81% (49%, 113%)	58% (23%, 94%)	60% (39%, 81%)
Specificity (95% CI)	86% (72%, 99%)	90% (74%, 105%)	85% (65%, 105%)	93% (86%, 100%)
PPV (95% CI)	65% (34%, 95%)	72% (33%, 111%)	54% (6%, 102%)	69% (42%, 95%)
NPV (95% CI)	89% (77%, 101%)	93% (81%, 106%)	87% (70%, 105%)	90% (81%, 98%)
**Diagnosing benign lesions**				
Sensitivity (95% CI)	67% (51%, 83%)	81% (63%, 100%)	63% (40%, 85%)	75% (65%, 85%)
Specificity (95% CI)	85% (71%, 100%)	95% (86%, 104%)	80% (54%, 106%)	79% (64%, 93%)
PPV (95% CI)	88% (75%, 101%)	98% (92%, 103%)	86% (66%, 106%)	90% (82%, 98%)
NPV (95% CI)	62% (41%, 83%)	68% (37%, 99%)	52% (20%, 83%)	54% (35%, 73%)

Abbreviations: CKD (chronic kidney disease), ESKD (end-stage kidney disease), PPV (positive predictive value), NPV (negative predictive value), CI (confidence interval).

## Data Availability

The data underlying this article cannot be shared publicly to protect the privacy of the individuals who consented to participate in the study. The data will be shared on reasonable request to the corresponding author.

## Supplementary Materials

The following supporting information can be downloaded at: https://www.mdpi.com/article/10.3390/jcm12206494/s1, Figure S1: A series of images demonstrating the flash replenishment imaging technique; Figure S2: Graphical user interface layout; Table S1: Characteristics of all enrolled patients; Table S2: Number, diagnosis, laterality, and size of analyzed lesions; Table S3: Inter-reader agreement on lesion characterization; Table S4: Contrast ultrasound data quality assessment of interpreted cases (*N* = 65) by reader; Video S1: An example of video data acquired using the flash replenishment imaging protocol in CPS mode, which shows both the kidney parenchyma and lesion (outlined by the cyan circle and labeled “lesion”).
