# Supplementary material for: The Performance of Flash Replenishment Contrast-Enhanced Ultrasound for the Qualitative Assessment of Kidney Lesions in Patients with Chronic Kidney Disease
Source: J Clin Med. 2023 Oct 12;12(20):6494. doi: 10.3390/jcm12206494 (PMC10607866; doi:10.3390/jcm12206494)
Supplement: Supplementary file 1 [file jcm-12-06494-s001.zip › jcm-2597993-supplementary.pdf]

Supplemental Materials

The performance of flash-replenishment contrast enhanced ultrasound for the qualitative assessment of kidney lesions in patients with chronic kidney disease

Rachel W. Walmer [1,*], Victor S. Ritter [2], Anush Sridharan [1,3], Sandeep K. Kasoji [1,4], Ersan Altun [5], Ellie Lee [5], Kristen Olinger [5], Sean Wagner [5], Roshni Radhakrishna [6], Kennita A. Johnson [1], W. Kimryn Rathmell [7], Bahjat Qaqish [2], Paul A. Dayton [1], and Emily H. Chang [6]

[1]Joint Department of Biomedical Engineering, University of North Carolina at Chapel Hill and North Carolina State University, Chapel Hill, NC, USA
[2]Department of Biostatistics, University of North Carolina at Chapel Hill, Chapel Hill, NC, USA
[3]Fox Chase Cancer Center, Philadelphia, PA, USA
[4]Triangle Biotechnology, Durham, NC, USA
[5]Department of Radiology, University of North Carolina at Chapel Hill, Chapel Hill, NC, USA
[6]Department of Medicine, University of North Carolina at Chapel Hill, Chapel Hill, NC, USA
[7]Department of Medicine, Vanderbilt University, Nashville, TN, USA

*Corresponding author

Corresponding Author: Rachel W. Walmer

Address: 10010 Mary Ellen Jones CB 7575, 116 Manning Drive, Chapel Hill, NC, 27599

Email: rachel.w.walmer@unc.edu

SUPPLEMENTAL FIGURES AND TABLES

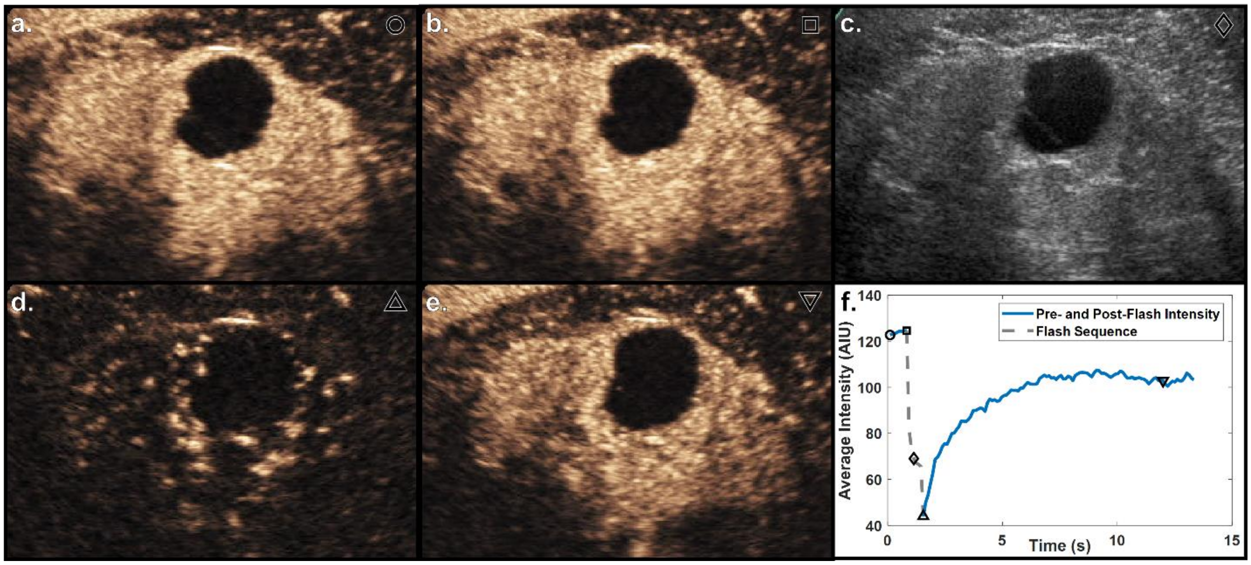

Figure S1. A series of images demonstrating the flash replenishment imaging technique. Symbols in the upper right-hand corner of a-e) correspond to their location on the graph of intensity vs. time in f). a) Contrast intensity after reaching steady state equilibrium via a constant infusion of Perflutren Lipid Microspheres, b) the intensity immediately prior (pre-flash) to initiating a high pressure pulse to clear contrast from the kidney, c) the high pressure pulse, or flash, is generated by briefly switching to color doppler imaging, d) the first frame (post-flash) after returning to a low-pressure contrast imaging mode, where contrast can be seen beginning to refill the kidney, e) the contrast intensity as it returns to the original steady state value, and f) a graph of intensity over time in the kidney which represents the flash replenishment imaging technique.

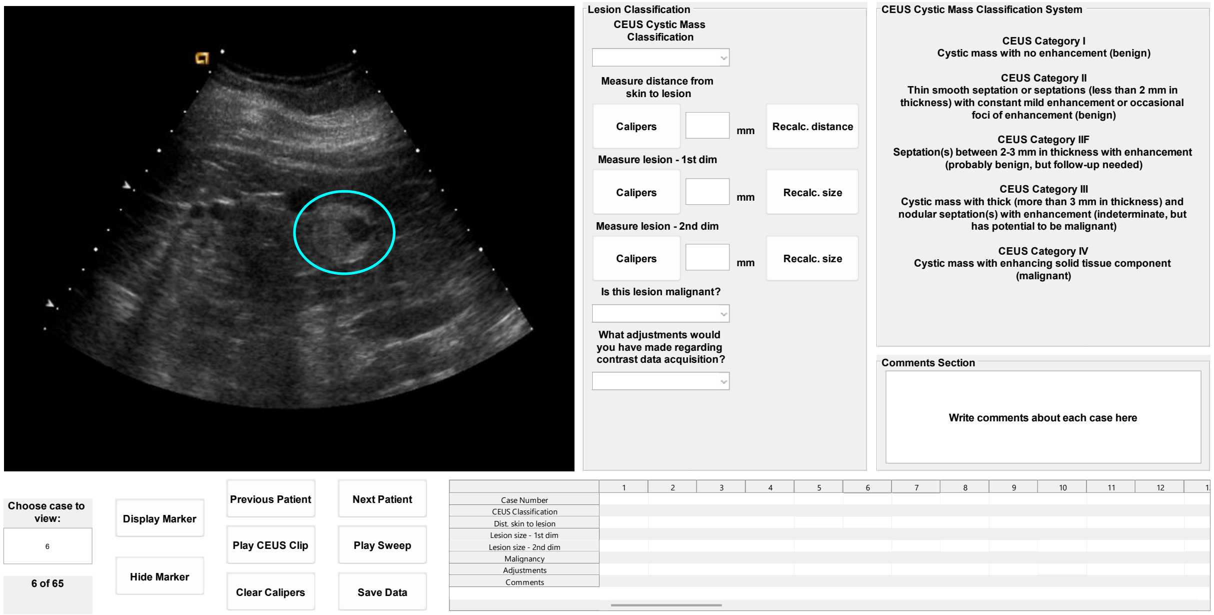

Figure S2. Graphical user interface layout. The reader interacts with the main screen to assess each lesion. The B-mode image is displayed on the left, with buttons that allow the user to move from patient to patient, change what is displayed, and externally play contrast videos. The middle panel contains drop-down question menus and buttons for taking measurements on the B-mode image. Definitions and a section for adding comments are located on the right. The table underneath the two panels populates with the responses as the reader completes each case.

Table S1. Characteristics of all enrolled patients

Patient Characteristics (N = 60)	N (%)
**Age (years)**	
Mean ± SD	60 ± 14
**Sex**	
Male	37 (61.7%)
Female	23 (38.3%)
**Race or Ethnicity**	
Black	30 (50.0%)
White	29 (48.3%)
Hispanic	1 (1.7%)
**CKD Stage**	
CKD II	9 (15%)
CKD III	18 (30%)
CKD IV	11 (18.3%)
CKD V	5 (8.3%)
ESKD	17 (28.3%)
**Initial Imaging Study**	
Non-contrast CT	3 (5.0%)
Contrast CT	2 (3.3%)
Contrast CT with renal mass protocol	12 (20%)
Non-contrast MRI	3 (5.0%)
Contrast MRI	3 (5.0%)
Conventional US	37 (61.7%)

Abbreviations: CKD (chronic kidney disease), ESKD (end-stage kidney disease), CT (computed tomography), MRI (magnetic resonance imaging), US (ultrasound)

Table S2. Number, diagnosis, laterality, and size of analyzed lesions

Lesion Information (N = 63)	N (%)
**Lesion Diagnosis**	
Clear cell RCC	2 (3.2%)
Acquired cystic disease RCC	4 (6.4%)
Papillary RCC	2 (3.2%)
Clear cell papillary RCC	2 (3.2%)
Tubulocystic RCC	1 (1.6%)
Oncocytoma	1 (1.6%)
Imaging Surveillance	
Stable: Regressed/no concerning features	40 (63.5%)
Suspicious: Stable, but persistent concerning features	8 (12.7%)
Progressed: Progressed features	3 (4.7%)
**Laterality of Lesion**[1]	
Left	30 (47.6%)
Right	33 (52.4%)
**Lesion Measurements by Imaging (cm^3, cm)**[2]	
Average Volume ± SD (range)	10.8 ± 14.3 (0.15-85.4)
Median Volume (IQR)	5.97 (11.4)
Average Minimum Diameter (range)	2.06 (0.60-4.40)
Average Maximum Diameter (range)	2.72 (0.80-6.90)

[1]Lesion laterality is indicated based on each individual lesion/case, regardless of if bilateral disease was present in patients with multiple lesions imaged for this study. [2]Some values are reported to two decimal places to retain a minimum of two significant figures for numbers other than exact numbers.
Abbreviations: RCC (renal cell carcinoma), IQR (interquartile range)

Table S3. Inter-reader agreement on lesion characterization

	**Reader 1**	**Malignant (n = 36*)**	**Indeterminate (n = 5*)**	**Benign (n = 22*)**	***P*-value**
**Reader 2**	Malignant	26 (72%)	2 (40%)	1 (4.5%)	0.2
	Indeterminate	10 (28%)	1 (20%)	1 (4.5%)	
	Benign	0 (0%)	2 (40%)	20 (91%)	
**Reader 3**	Malignant	27 (75%)	1 (20%)	2 (9.1%)	0.3
	Indeterminate	6 (17%)	1 (20%)	2 (9.1%)	
	Benign	3 (8.3%)	3 (60%)	18 (82%)	
**Reader 4**	Malignant	27 (75%)	1 (20%)	4 (18%)	0.4
	Indeterminate	6 (17%)	4 (80%)	0 (0%)	
	Benign	3 (8.3%)	0 (0%)	18 (82%)	

	**Reader 2**	**Malignant (n = 29*)**	**Indeterminate (n = 12*)**	**Benign (n = 22*)**	***P*-value**
**Reader 3**	Malignant	23 (79%)	6 (50%)	1 (4.5%)	0.7
	Indeterminate	4 (14%)	4 (33%)	1 (4.5%)	
	Benign	2 (6.9%)	2 (17%)	20 (91%)	
**Reader 4**	Malignant	26 (90%)	4 (33%)	2 (9.1%)	0.8
	Indeterminate	2 (6.9%)	6 (50%)	2 (9.1%)	
	Benign	1 (3.4%)	2 (17%)	18 (82%)	

	**Reader 3**	**Malignant (n = 30*)**	**Indeterminate (n = 9*)**	**Benign (n = 24*)**	***P*-value**
**Reader 4**	Malignant	25 (83%)	5 (56%)	2 (8.3%)	0.6
	Indeterminate	4 (13%)	2 (22%)	4 (17%)	
	Benign	1 (3.3%)	2 (22%)	18 (75%)	

*Percentages totaled by column, for each individual reader compared to the section header

Table S4. Contrast ultrasound data quality assessment of interpreted cases (N = 65) by reader

Suggestion	Reader 1	Reader 2	Reader 3	Reader 4
No suggested changes	48	24	47	48
Higher contrast dose	3	13	3	4
Lower contrast dose	0	1	0	0
Longer imaging	1	0	0	2
Adjust gain	1	2	6	2
Different view	11	21	8	1
Other major adjustments	0	0	0	3
Other minor adjustments	1	4	1	5
